# The chemopreventive properties of chlorogenic acid reveal a potential new role for the microsomal glucose-6-phosphate translocase in brain tumor progression

**DOI:** 10.1186/1475-2867-6-7

**Published:** 2006-03-27

**Authors:** Anissa Belkaid, Jean-Christophe Currie, Julie Desgagnés, Borhane Annabi

**Affiliations:** 1 Laboratoire d'Oncologie Moléculaire, Département de Chimie, Centre BIOMED, Université du Québec à Montréal, Montreal, Quebec, Canada

## Abstract

**Background:**

Chlorogenic acid (CHL), the most potent functional inhibitor of the microsomal glucose-6-phosphate translocase (G6PT), is thought to possess cancer chemopreventive properties. It is not known, however, whether any G6PT functions are involved in tumorigenesis. We investigated the effects of CHL and the potential role of G6PT in regulating the invasive phenotype of brain tumor-derived glioma cells.

**Results:**

RT-PCR was used to show that, among the adult and pediatric brain tumor-derived cells tested, U-87 glioma cells expressed the highest levels of G6PT mRNA. U-87 cells lacked the microsomal catalytic subunit glucose-6-phosphatase (G6Pase)-α but expressed G6Pase-β which, when coupled to G6PT, allows G6P hydrolysis into glucose to occur in non-glyconeogenic tissues such as brain. CHL inhibited U-87 cell migration and matrix metalloproteinase (MMP)-2 secretion, two prerequisites for tumor cell invasion. Moreover, CHL also inhibited cell migration induced by sphingosine-1-phosphate (S1P), a potent mitogen for glioblastoma multiform cells, as well as the rapid, S1P-induced extracellular signal-regulated protein kinase phosphorylation potentially mediated through intracellular calcium mobilization, suggesting that G6PT may also perform crucial functions in regulating intracellular signalling. Overexpression of the recombinant G6PT protein induced U-87 glioma cell migration that was, in turn, antagonized by CHL. MMP-2 secretion was also inhibited by the adenosine triphosphate (ATP)-depleting agents 2-deoxyglucose and 5-thioglucose, a mechanism that may inhibit ATP-mediated calcium sequestration by G6PT.

**Conclusion:**

We illustrate a new G6PT function in glioma cells that could regulate the intracellular signalling and invasive phenotype of brain tumor cells, and that can be targeted by the anticancer properties of CHL.

## Background

The beneficial effects of dietary polyphenols on human health have been widely assumed to act through various biological effects such as free radical scavenging, metal chelation, modulation of enzymatic activity and altering signal transduction pathways [1-3]. Epidemiological studies have also highlighted the association between the consumption of polyphenol-rich food and beverages and the prevention of various human diseases [4,5]. Among these polyphenols, the antitumor activities of flavonoids as well as the inhibition of carcinogenesis by polyphenols has revealed properties beneficial for the use of nutraceuticals in cancer therapy [6-8]. Among the sources of these anticancer polyphenols, modern phytochemical research shows that tea contains a large number of plant secondary metabolites exhibiting different chemical structures such as amino acids, catechins, purine alkaloids, and chlorogenic acid (CHL), and where each group of compounds possesses some unique biological properties [9]. While green tea catechins have now been established as having chemopreventive effects [10,11], the impact of CHL, to which have been attributed possible cancer chemoprevention properties, is not well understood [12,13]. Interestingly, CHL inhibition of matrix metalloproteinase (MMP)-9 secretion, an MMP known to be involved in tumor cell invasion and metastasis, was recently reported but the anti-cancer intracellular molecular mechanisms through which CHL effects occur are remained unexplored [14]. This property, however, adds up to CHL's antioxydant and anti-inflammatory properties [15,16].

CHL derivatives have been shown to selectively inhibit endoplasmic reticulum (ER) glucose-6-phosphate (G6P) transport, and hence microsomal glucose-6-phosphatase (G6Pase) activity both in isolated microsomes [17] and *in vivo *[18,19]. CHL is a specific, reversible, competitive inhibitor of G6PT [20], and it has no effect on the intraluminal, G6P-hydrolytic subunit [21]. In intact cells, the CHL derivative and G6PT inhibitor S3483 was found to inhibit G6P transport in microsomes isolated from polymorphonuclear neutrophils (PMN) and from differentiated promyelocytic HL-60 cells [22]. Interestingly, the PMN phenotype in glycogen storage disease (GSD) type 1b, a clinical condition where the G6PT gene or protein is defective [22,23], includes diminution in several processes such as respiratory burst, chemotaxis, phagocytosis and calcium signalling [24-26]. Alterations in several biochemical parameters – glucose phosphorylation, calcium mobilization, and hexose uptake and transport – have been described as possible mechanisms through which the G6PT functional defects may be involved [27-29]. Since cells such as PMN have no detectable G6Pase activity, G6PT must play a role different from that exerted in the liver, for instance, where it is functionally coupled to the G6Pase enzyme. Moreover, G6PT functions have never been investigated in brain tumor-derived cells. It has been hypothesized that G6PT might function as a G6P receptor/sensor [23] or that it could favor calcium sequestration in the ER lumen [30]. Such roles have not yet been evidenced, neither the alternate potential G6PT-regulated cellular functions explored.

In the present work two topics have been addressed. Does functional inhibition of G6PT regulate any brain tumor-derived cells' tumorigenic properties, such as MMP-mediated extracellular matrix (ECM) hydrolytic activity or cell migration ? If so, can a connection be made between G6PT functions and a role in intracellular signalling that regulates the invasive phenotype ? Inhibition of the microsomal G6PT functions was modeled by the addition of CHL, which is a highly specific inhibitor of G6PT [31], while upregulation of G6PT was performed through cDNA transient transfection. The results demonstrate that G6PT may regulate the brain tumor-derived invasive phenotype by controlling intracellular signalling that leads to cell migration. Moreover, we provide the first molecular rationale for the anticancer properties of CHL in the regulation of MMP secretion.

## Results

### U-87 glioma cells express the highest levels of G6PT transcript among brain tumor-derived cell lines

**Figure 1:**
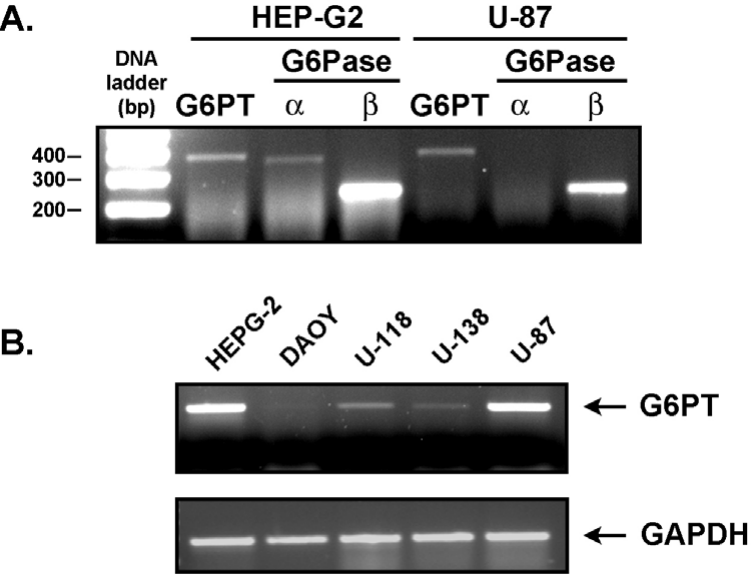
**U-87 glioma cells express G6PT and G6Pase-β transcripts with the highest G6PT transcript levels among brain tumor-derived cell lines**. Several brain tumor-derived cell lines (glioma : U-87, U-118, U-138; medulloblastoma : DAOY) were cultured until cells reached approximately 90% confluence. Total RNA was extracted and RT-PCR performed in order to generate the cDNA that reflects the gene expression levels of G6PT (380 bp), G6Pase-α (360 bp), and G6Pase-β (236 bp) as described in the methods section. HEP-G2 hepatoma cells were used as positive controls for the presence of all three genes. GAPDH gene expression was used as an internal control for each cell line tested.

Gene expression levels of the microsomal glucose-6-phosphate transporter (G6PT), as well as of the two glucose-6-phosphatase α and β isoforms, were first assessed. Total RNA was extracted from HEP-G2 hepatoma and U-87 glioma cells, and then RT-PCR was performed as described in the Methods section. As expected for a cell line derived from a neoglucogenic tissue, we show that HEP-G2 cells express all three components of the G6Pase system with G6Pase-β being predominant amongst these three (Fig. 1a). RT-PCR was also performed on RNA extracted from U-87 glioma cells. In contrast to HEP-G2, only G6PT and G6Pase-β transcripts were significantly expressed, with very low to undetectable G6Pase-α present (Fig. 1a). This is in agreement with previous reports demonstrating the lack of G6Pase-α expression in brain-derived cells [32]. We also monitored the levels of G6PT in a brain tumor cell line derived from a pediatric medulloblastoma (DAOY), as well as in the U-118 and U-138 glioma cell lines. Interestingly, among the adult brain tumor-derived cell lines, U-87 glioma cells expressed the highest level of G6PT (Fig. 1b), while DAOY cells expressed very low to undetectable G6PT transcripts. This suggests that G6PT expression in brain tumor cells may be regulated during development.

### The G6PT inhibitor chlorogenic acid reduces proMMP-2 secretion in U-87 glioma cells

**Figure 2:**
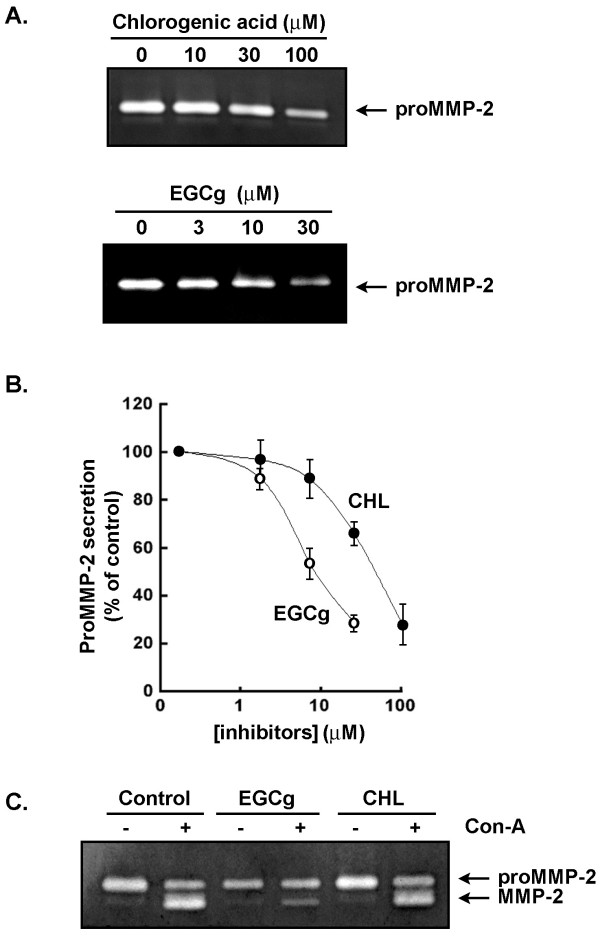
**The G6PT inhibitor chlorogenic acid reduces proMMP-2 secretion in U-87 glioma cells**. (A) U-87 glioma cells were serum-starved in the presence of increasing concentrations of CHL and of the green tea catechin EGCg for 18 hrs. Conditioned media was collected and proMMP-2 gelatinolytic activity assessed by zymography as described in the methods section. (B) The extent of proMMP-2 hydrolytic activity was quantified by scan densitometry and is the average ± SEM of three independent experiments. (C) The effect of CHL and EGCg on MT1-MMP functions was also monitored. U-87 cells were cultured in the presence of an exogenous source of proMMP-2 in the presence of 10 μg/ml concanavalin-A, a MT1-MMP-mediated inducer of proMMP-2 activation, and of EGCg or CHL. The extent of proMMP-2 activation was assessed by gelatin zymography.

We next assessed the potential functions that G6PT may affect in the invasive phenotype of U-87 glioma cells. Based on the assumption that CHL is able to efficiently penetrate inside the cells [33], U-87 glioma cells were treated with different concentrations of CHL and the secretion of matrix metalloproteinase (MMP)-2 was monitored by zymography. CHL significantly inhibited latent proMMP-2 secretion (Fig. 2a) and another tea-derived compound, epigallocatechin gallate (EGCg), was just as efficient (Fig. 2a and 2b). CHL was found not to induce any early/late apoptotic or necrotic processes in U-87 glioma cells as assessed by Annexin-V/Propidium iodine double staining and flow cytometry (not shown). Since EGCg has also been reported to inhibit membrane type (MT)1-MMP from activating the latent proMMP-2 in glioblastoma cells [34], we further assessed the possible effects of CHL on MT1-MMP-mediated proMMP-2 activation. Cells were incubated with an exogenous source of proMMP-2 and were treated (or not) with concanavalin-A in order to trigger proMMP-2 activation [35]. While EGCg clearly inhibited MT1-MMP-mediated activation of proMMP-2, CHL had no such inhibitory effect (Fig. 2c).

### Chlorogenic acid inhibits G6PT-mediated U-87 glioma cell migration

**Figure 3:**
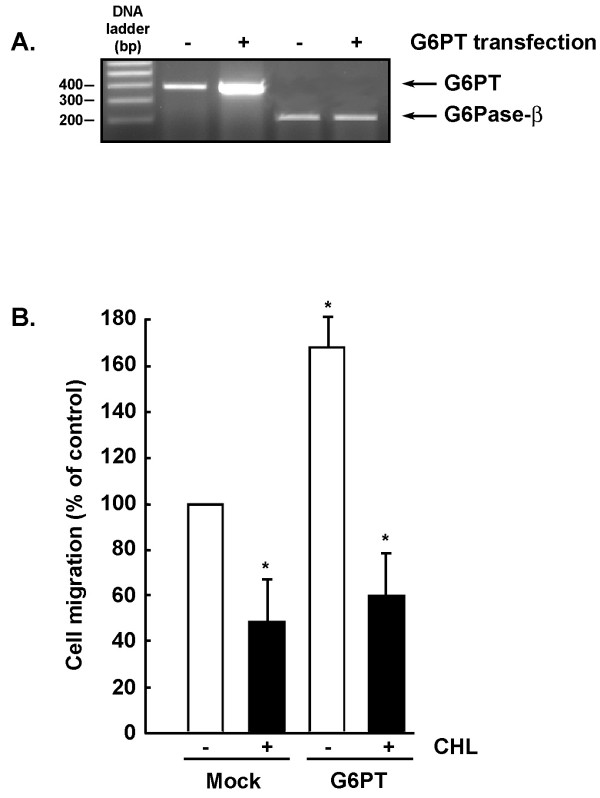
**G6PT regulates U-87 glioma cells migration**. U-87 glioma cells were transfected with a cDNA plasmid encoding G6PT [36] as described in the methods section. (A) Total RNA was extracted from Mock or G6PT-transfected cells (Tx) and RT-PCR was independently performed for G6PT and G6Pase-β gene expression. (B) Basal cell migration was assessed in Mock and G6PT-transfected U-87 cells as described in the Methods section in the presence or absence of 100 μM CHL. Six independent experiments were performed and the average of more than 10 microscopic fields for each experimental condition shown with SEM.

The role of G6PT in U-87 glioma cell migration was next assessed. Cells were transfected, or not (Mock), with G6PT cDNA [36] and basal cell migration assay was performed on gelatin-coated filters using modified Boyden chambers as described in the Methods section. Transfection of G6PT cDNA specifically increased G6PT transcript levels, while levels of G6Pase-β remained unaffected (Fig. 3a). Interestingly, the sole effect of G6PT overexpression was to increase the basal migration of U-87 glioma cells (Fig. 3b, white bars), while CHL inhibited both basal and G6PT-induced cell migration (Fig. 3b, black bars). This suggests that G6PT functions may, in part, regulate the invasive phenotype of U-87 glioma cells.

### Chlorogenic acid inhibits sphingosine-1-phosphate-induced U-87 glioma cells migration

**Figure 4:**
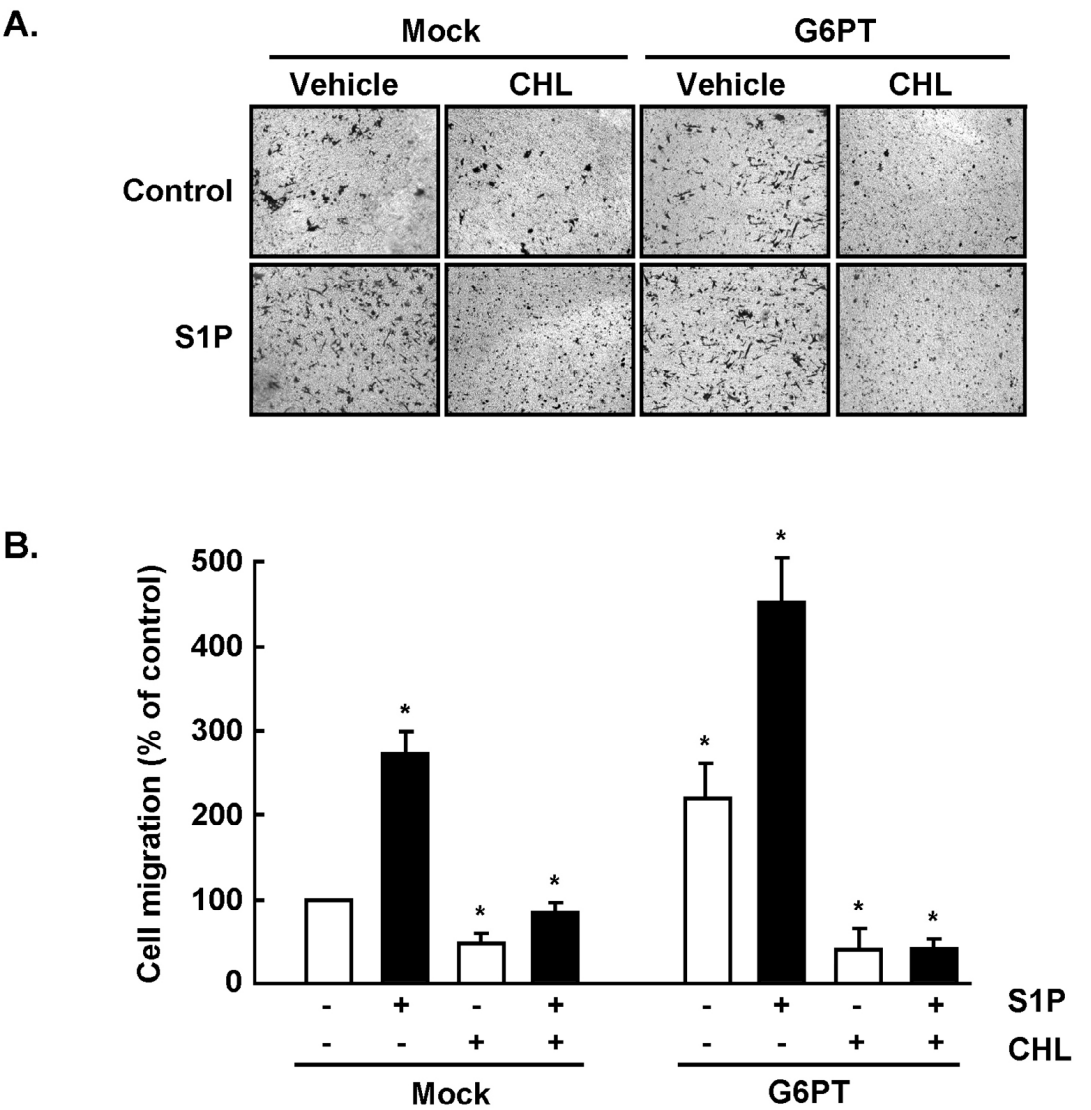
**Chlorogenic acid inhibits sphingosine-1-phosphate-induced U-87 glioma cells migration**. (A) Control (Mock) or G6PT-transfected U-87 glioma cells were seeded on gelatin-coated filters and cell migration was assessed in the presence or absence of 100 μM CHL, 1 μM S1P, or a combination of both. (B) Quantification was performed as described in the Methods section and is representative of 6 independent experiments. The average of more than 10 microscopic fields for each experimental condition is shown with SEM.

Sphingosine-1-phosphate (S1P) is a bioactive lipid which is present at high levels in brain tissue [36] and which acts as a potent mitogen for glioblastoma multiform cells [38]. More recently, S1P was shown also to potently enhance the *in vitro *motility of glioblastoma cells [39]. We thus assessed whether CHL could interfere with both basal and S1P-induced cell migration in U-87 cells, and if it could antagonize the G6PT-mediated regulation of cell migration. Control (Mock) or G6PT-transfected U-87 glioma cells were harvested and seeded on top of gelatin-coated filters as described in the Methods section. Cell migration then occurred in the presence of either S1P, CHL, or a combination of both. Representative images of stained cells that had migrated through the filters are shown (Fig. 4a). We observed that S1P induced basal cell migration by 2.6-fold, and that CHL was able to inhibit both the basal and S1P-induced cell migration (Fig. 4b, Mock). When cells were transiently transfected with the G6PT cDNA, basal cell migration increased by approximately 2-fold, in accordance with Fig. 3b. Interestingly, the effect of S1P was additive to that of the overexpression of G6PT, but CHL efficiently antagonized migration that was induced either by G6PT overexpression or by S1P in G6PT-transfected cells (Fig. 4b, G6PT). These results suggest that G6PT potentially regulates the intracellular signalling that triggers basal and S1P-mediated cell migration, and that functional inhibition of this signalling by CHL could explain some of its chemopreventive effects at the molecular level.

### Chlorogenic acid inhibits sphingosine-1-phosphate-induced ERK phosphorylation in U-87 glioma cells

**Figure 5:**
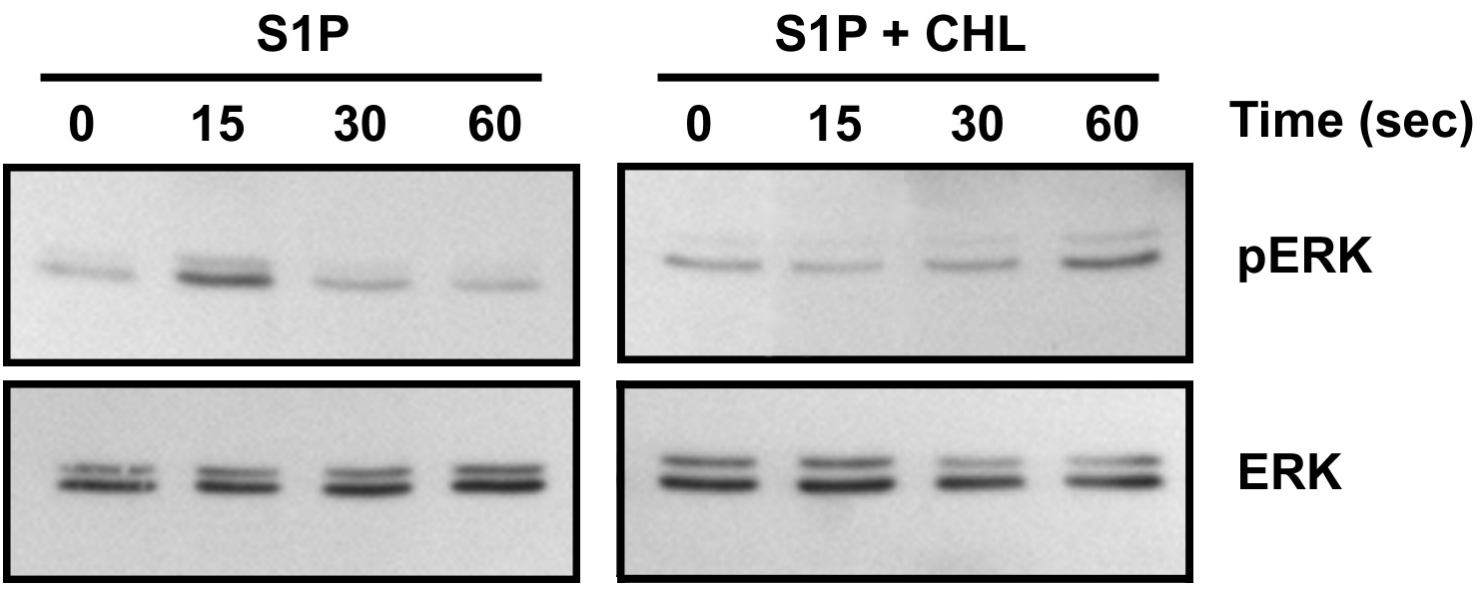
**Chlorogenic acid inhibits sphingosine-1-phosphate-induced ERK phosphorylation in U-87 glioma cells**. Serum-starved U-87 glioma cells were pretreated with for 30 minutes with 100 μM CHL. One μM S1P was then added to the media and incubation performed for 15, 30, and 60 seconds. Cells were then rapidly harvested and lysates prepared as described in the Methods section. The extent of ERK phosphorylation was monitored by immunoblotting using antibodies against p-ERK and total ERK.

S1P is thought to trigger intracellular signalling, in part, through the MAPK pathway and the release of intracellular calcium pools [38,40]. We thus monitored the extent of ERK phosphorylation that is triggered by S1P in U-87 glioma cells and whether CHL could interfere with that process. S1P triggered a rapid phosphorylation of ERK within the first 15 seconds of incubation (Fig. 5, left panel). Interestingly, pre-incubation of the cells with 100 μM CHL for 30 minutes prevented that rapid phosphorylation of ERK and even delayed it until a time of 60 seconds (Fig. 5, right panel). This inhibitory effect of CHL on S1P-induced ERK phosphorylation suggests that intracellular inhibition of microsomal G6PT activity may antagonize crucial events taking place in the ER.

### Intracellular ATP-depleting agents inhibit proMMP-2 secretion and antagonize S1P-induced ERK phosphorylation in U-87 glioma cells

**Figure 6:**
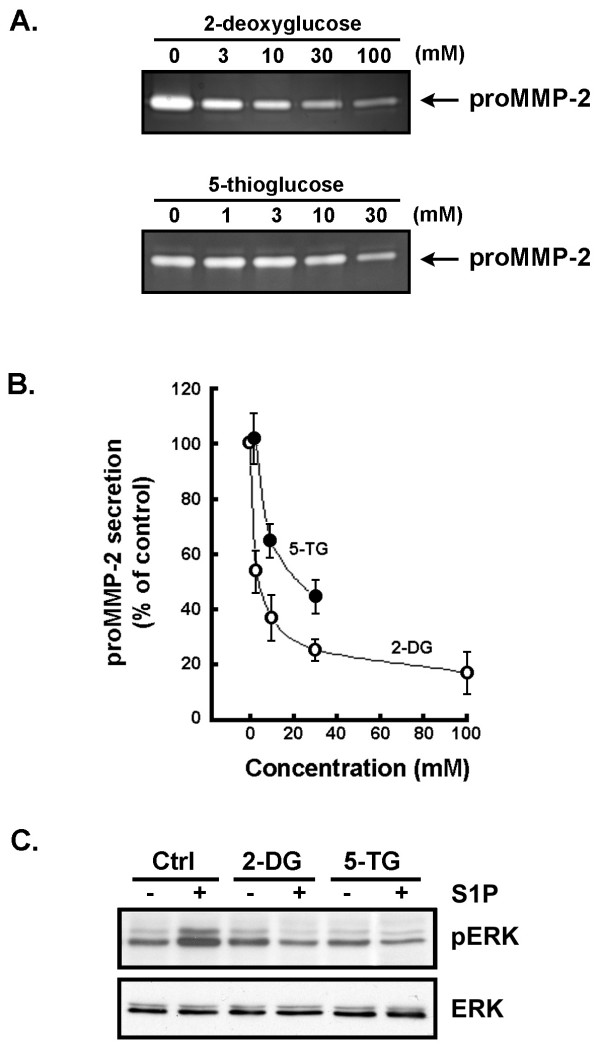
**Intracellular ATP-depleting agents inhibit proMMP-2 secretion and antagonize S1P-induced ERK phosphorylation in U-87 glioma cells**. (A) Serum-starved U-87 glioma cells were treated for 18 hrs with individual concentrations of 2-deoxyglucose or 5-thioglucose, two well established ATP-depleting agents [61]. Conditioned media were collected and the hydrolytic activity of proMMP-2 was assessed using gelatin-zymography as described in the Methods section. (B) The extent of proMMP-2 hydrolytic activity was quantified by scan densitometry in 5-TG- (black circles) and 2-DG- (white circles) treated cells, and is the average ± SEM of three independent experiments. (C) Serum-starved U-87 glioma cells were pretreated for 30 minutes with 100 mM 2-deoxyglucose (2-DG) or 30 mM 5-thioglucose (5-TG). One μM S1P was then added to the media for 15 seconds. Cells were then rapidly harvested and lysates prepared as described in the Methods section.

Besides its classical role in recognizing and translocating G6P from the cytoplasm into the lumen of the ER, G6PT has been attributed a possible role in regulating calcium flux responses [41,42]. Such a role in regulating intracellular calcium pools was elegantly demonstrated in neutrophils isolated from G6PT-deficient mice [43]. Interestingly, a functional link has been proposed between G6P and calcium such that cytoplasmic G6P is thought to enhance ATP-dependent sequestration of calcium in the ER [30]. In light of this, we sought to investigate the effects of the ATP-depleting agents and synthetic analogs of glucose, the glucose 5-thioglucose (5-TG) and the 2-deoxy-d-glucose (2-DG), on proMMP-2 secretion and S1P-induced ERK phosphorylation. U-87 glioma cells were serum-starved and treated with different concentrations of 2-DG and 5-TG. Conditioned media was isolated and the activity of secreted proMMP-2 was measured. ProMMP-2 secretion was significantly decreased in both 2-DG- and 5-TG-treated cells (Fig. 6a). Half-maximal inhibition constants (IC_50_) were calculated to be 2 mM and 19 mM, respectively, for 2-DG and 5-TG (Fig. 6b). S1P-induced ERK phosphorylation was also monitored in 2-DG- and 5-TG pre-treated cells, and both ATP-depleting agents were able to inhibit S1P-induced ERK phosphorylation (Fig. 6c). Although these data are indirect, altogether they suggest that the inhibition of microsomal G6PT functions (inhibition of ER G6P uptake or of ATP-dependent calcium sequestration) leads to decreased proMMP-2 secretion and a potential decrease in calcium-mediated intracellular signalization.

## Discussion

As part of our continuing search for potential anticancer drug candidates, we pay great interest to natural products for their potential anticancer activities. As such, among the sources of anticancer polyphenols in tea constituents, CHL has recently caught our attention as it has been attributed chemopreventive properties [12-14]. Although CHL is known as the most potent inhibitor of G6PT, no study has yet demonstrated a role for G6PT in regulating tumor progression and metastasis. Our study now provides a molecular rationale to the anticancer properties of CHL in highly invasive brain tumor-derived U-87 glioblastoma cells. Indeed, we highlight a new and underestimated function for the microsomal G6PT as a potential regulator of cancer cells invasive phenotype. Besides its classical role in regulating the rate limiting step of G6P transport, G6PT specific functional inhibition by CHL even becomes more relevant when one considers G6PT's potential role in regulating calcium-mediated signalling. Such intracellular transduction events are known to regulate cancer cells proliferation, cell cycle division, ECM degradation, and response to growth factors. We now suggest that new cellular processes such as cell invasion, intracellular signal transduction in response to growth factors, and secretion of MMP may collectively be regulated in part through G6PT-mediated ER functions.

Interestingly, we extensively investigated and demonstrated that EGCg, another naturally occurring green tea-derived polyphenol with anticancerous properties similar to those we describe for CHL, can also efficiently target the brain tumor invasive phenotype by inhibiting RhoA/Rock-mediated intracellular cell signalling [44]. Moreover, in light of recent studies which have shown that EGCg could also serve as an ionizing radiation (IR) enhancer against cancer cell lines [45], we have further shown that EGCg pretreatment of glioma cells prior to IR can reverse the cytoprotective effect provided through the expression of prosurvival proteins such as Survivin [46]. Functional inhibition of G6PT by either CHL or by the ATP-depleting agents 2-DG/5-TG could thus provide a molecular rationale for the role that G6PT may play in the intracellular signalling regulating the high-grade radioresistant glioma cells.

One important implication of our study is its potential impact on targeting G6PT functions in radiotherapeutic modalities. The failure of radiotherapy in cerebral gliomas is primarily due to the diffusely infiltrating nature of the tumor and the presence of a hypoxic, repair-proficient and intrinsically radioresistant subpopulation of cells. Since the chemopreventive properties of both the IR-cell sensitizing agent EGCg [46] and of CHL molecules [this study] are similarly and efficiently able to inhibit cell migration and MMP-secretion of glioblastoma cells, it is tempting to suggest that CHL may also be used in synergy with radiotherapeutic modalities. Enhanced glucose usage *in vitro *as well an *in vivo*, which correlates with the degree of malignancy and with poor prognosis, has been demonstrated in glioma tumors [52-54]. Moreover, *in vitro *studies in established glioma cell lines showed that the presence of 2-DG for a few hours after irradiation could increase radiation damage significantly and that radiosensitization was higher under conditions of reduced respiratory metabolism [55]. Finally, 2-DG is known to inhibit glycolytic energy ATP production [56] and has been tested in multiple studies for possible application as an anticancer or antiviral therapeutic [57-59]. Such observations provide another molecular rationale for the means by which CHL, 2-DG and 5-TG that we report in the present study may inhibit G6PT functions, and that we have shown to be significantly expressed in U-87 glioblastoma cells.

Poorly differentiated and rapidly growing malignant tumors are generally characterized by higher rates of glucose usage and glycolysis as compared to corresponding normal tissues [47,48]. As such, these fundamental differences in the glucose metabolism of transformed and normal cells form the basis for the noninvasive detection and grading of tumors by positron emission tomography (PET) using tracer nanomolar doses of [^18^F]-2-fluoro-deoxyglucose ([^18^F]FDG), a nonmetabolizable analog of glucose that enters the cell by the same membrane transport mechanism as does glucose. Measurements of glucose uptake into human tumors by FDG-PET suggest that glucose uptake may directly correlate with the degree of malignancy and treatment resistance/poor prognosis [49]. The precise molecular mechanisms underlying this correlation remain to be elucidated. Interestingly, the use of [^18^F]FDG was also employed in the monitoring of adenovirus-mediated GSD-1a correction [50], a disease characterized by a defect in the glucose-6-phosphatase catalytic subunit (G6Pase-α). Therefore, similar to the non-metabolizable glucose analogue 2-DG, one can expect pharmacological doses of FDG to block glycolysis and to cause depletion of ATP. Interestingly, it has been shown that the killing of neoplastic cells by cytostatic drugs is associated with decreased ATP content and FDG uptake. This indicates that FDG uptake is closely linked with ATP production and that not only ATP but also FDG could be used to study drug effects *in vitro *[51].

We show that inhibiting G6PT functions by CHL or by ATP-depleting agents in brain-tumor-derived cells may result in decreased invasiveness. Moreover, because cancer cells frequently display high rates of aerobic glycolysis, in comparison to their nontransformed counterparts, the recently published hypoglycemic impact of CHL [60], combined with the possible applications of 2-DG in anticancer therapies, further supports the theory that inhibiting G6PT functions in cancer cells could decrease tumor progression. G6PT deficiencies lead to GSD type-Ib and chronic neutropenia because of impaired glucose homeostasis [41,42], altered neutrophil chemotaxis and calcium flux [43], and induction of cell apoptosis [22]. Concomitantly, CHL was recently found to inhibit human hepatocellular carcinoma cell line proliferation [14] and to induce apoptosis of chronic myelogenous leukemia (CML) cell lines and primary cells from CML patients [62]. Alternate cellular targets of CHL in glioma cells can not be excluded as its most extensively studied effects were attributed to its metal chelator, and that its anti-oxidative properties highlighted a role for ERK in oxidative stress-induced glioma cell death [63]. Although this remains to be investigated, it thus becomes tempting to hypothesis that the anti-oxidative properties of CHL may somehow prevent or protect from oxidative stress-induced cell death. Studies on the G6PT functions and contribution in neurodegenerative diseases may shed some clues as to its role and modulation by oxidative stress.

In summary, our study evidences that G6PT triggers cancer cell migration, and that CHL antagonized growth factors-induced calcium mobilization and ERK phosphorylation. The sum of these experimental evidences leads us to postulate a new role for G6PT in cancer cell signalling, and suggests that efficient inhibition of the G6PT functions will impact on the invasive and metastatic phenotype of circulating cancer cells whether they derive from solid tumors or from malignant clonal disorder of hematopoietic stem cells such as CML.

## Methods

### Materials

Agarose, (-)-epigallocatechin 3-gallate (EGCg), sodium dodecylsulfate (SDS), gelatin, and bovine serum albumin (BSA) were purchased from Sigma (Oakville, ON). The TRIzol reagent was from Life Technologies (Gaithersburg, MD). FUGENE-6 transfection reagent was from Roche Diagnostics Canada (Laval, QC).

### Cell culture and cDNA transfection method

The U-87, U-118 and U-138 glioma cell lines, the DAOY medulloblastoma cell line and the HEP-G2 hepatoma cell line were purchased from American Type Culture Collection and cultured in their recommended media. Specifically, U-87 cells were maintained in Eagle's Minimum essential medium (MEM) containing 10% (v/v) fetal bovine serum (FBS) (HyClone Laboratories, Logan, UT), 2 mM glutamine, 100 units/ml penicillin and 100 μg/ml streptomycin, and were cultured at 37°C under a humidified atmosphere containing 5% CO_2_. The G6PT plasmid was validated and generously provided by Dr Christopher Newgard [35]. U-87 cells were transiently transfected with the cDNA construct using the non-liposomal formulation FUGENE-6 transfection reagent. Transfection efficiency was confirmed by RT-PCR. All experiments involving these cells were performed 36 hrs following transfection. Mock transfections of U-87 cultures with pcDNA (3.1+) expression vector alone were used as controls.

### Total RNA isolation and reverse transcriptase-polymerase chain reaction (RT-PCR) analysis

Total RNA was extracted from cultured monolayers of U-87 cells using the TRIzol reagent. One microgram of total RNA was used for first strand cDNA synthesis followed by specific gene product amplification with the One-Step RT-PCR Kit (Invitrogen, Burlington, ON). Primers for G6Pase-α (forward : 5'-TTCAGCCACATCCACAGCATC-3', reverse : 5'-GGGGTTTCAAGGAGTCAAAGACG-3'), for G6Pase-β (forward : 5'-ACTCTTCCTGACTTCTTGTGTGCC-3', reverse : 5'-TTGCCTTTGCTCTTTGGGGG-3') and for G6PT (forward : 5'-CAGGGCTATGGCTATTATCGCAC-3', reverse : 5'-ATGGCTCAAACCACTTCCGCAG-3') were all derived from human sequences. Glyceraldehyde-3-phosphate dehydrogenase (GAPDH) cDNA amplification was used as an internal house-keeping gene control. PCR conditions were optimized so that the gene products were examined at the exponential phase of their amplification and the products were resolved on 1.5% agarose gels containing 1 μg/ml ethidium bromide.

### Gelatin zymography

Gelatin zymography was used to assess the extent of MMP-2 activity. Briefly, an aliquot (20 μl) of the culture medium was subjected to SDS-PAGE in a gel containing 0.1 mg/ml gelatin. The gels were then incubated in 2.5% Triton X-100 and rinsed in nanopure distilled H_2_O. Gels were further incubated at 37°C for 20 hrs in 20 mM NaCl, 5 mM CaCl_2_, 0.02% Brij-35, 50 mM Tris-HCl buffer, pH 7.6, then stained with 0.1% Coomassie Brilliant blue R-250 and destained in 10% acetic acid, 30% methanol in H_2_O. Gelatinolytic activity was detected as unstained bands on a blue background. The exogenous source of proMMP-2, used to assess MT1-MMP-mediated activation of proMMP-2 by concanavalin-A, was isolated from 48 hrs serum starved-U87 glioma cells. This conditioned media contains high levels of latent proMMP-2 as well as TIMP-2 necessary to for the ternary complex with MT1-MMP and to monitor any potential MT1-MMP-mediated activation of proMMP-2 activation.

### Immunoblotting procedures

Proteins from control and treated cells were separated by SDS-polyacrylamide gel electrophoresis (PAGE). After electrophoresis, proteins were electrotransferred to polyvinylidene difluoride membranes which were then blocked overnight at 4°C with 5% non-fat dry milk in Tris-buffered saline (150 mM Tris, 20 mM Tris-HCl, pH 7.5) containing 0.3% Tween-20 (TBST). Membranes were further washed in TBST and incubated with the primary antibodies (1/1,000 dilution) in TBST containing 3% bovine serum albumin, followed by a 1 hr incubation with horseradish peroxidase-conjugated anti-rabbit IgG (1/10,000 dilution) in TBST containing 5% non-fat dry milk. Immunoreactive material was visualized by enhanced chemiluminescence (Amersham Biosciences, Baie d'Urfée, QC).

### Cell migration assay

Cells were dislodged after brief trypsinization, washed extensively and resuspended in DMEM at a concentration of 10^6 ^cells/ml [35]. Cells (7 × 10^5^) were then dispersed onto 1 mg/ml gelatin/PBS-coated chemotaxis filters (Costar; 8-μm pore size) within Boyden chamber inserts. Migration proceeded for 3 h at 37°C in 5% CO_2_. Cells that had migrated to the lower surface of the filters were fixed with 10% formalin phosphate, colored with 0.1% crystal violet/20% MeOH and counted by microscopic examination. The average number of migrating cells per field was assessed by counting at least four random fields per filter using Northern Eclipse software. Data points indicate the mean obtained from three separate chambers within one representative experiment.

### Statistical data analysis

Data are representative of three or more independent experiments. Statistical significance was assessed using nonparametric one-way ANOVA with GraphPad Prism Version 4.0. Probability values of less than 0.05 were considered significant, and an asterisk (*) identifies such significance in each figure.

## Abbreviations

ATP, adenosine triphosphate; CHL, chlorogenic acid; 2-DG, 2-deoxy-d-glucose; ECM, extracellular matrix; EGCg, epigallocatechin-(3)-gallate; ER, endoplasmic reticulum; ERK, extracellular signal-regulated protein kinases; G6P, glucose-6-phosphate; G6Pase, glucose-6-phosphatase; G6PT, G6P translocase; GSD, glycogen storage disease; IR, ionizing radiation; MMP, matrix metalloproteinase; 5-TG, 5-thioglucose.
